# How perceptions of autonomy relate to beliefs about inequality and fairness

**DOI:** 10.1371/journal.pone.0244387

**Published:** 2021-01-13

**Authors:** Abraham Aldama, Cristina Bicchieri, Jana Freundt, Barbara Mellers, Ellen Peters

**Affiliations:** 1 Center for Social Norms and Behavioral Dynamics, University of Pennsylvania, Philadelphia, PA, United States of America; 2 Department of Philosophy, Department of Psychology, and Center for Social Norms and Behavioral Dynamics, University of Pennsylvania, Philadelphia, PA, United States of America; 3 Chair of Industrial Economics, University of Fribourg, Fribourg, Switzerland; 4 Department of Psychology and Wharton School of Business, University of Pennsylvania, Philadelphia, PA, United States of America; 5 Center for Science Communication Research and School of Journalism and Communication, University of Oregon, Eugene, OR, United States of America; Ball State University, UNITED STATES

## Abstract

Although inequality in the US has increased since the 1960s, several studies show that Americans underestimate it. Reasons include overreliance on one’s local perspective and ideologically-motivated cognition. We propose a novel mechanism to account for the misperceptions of income inequality. We hypothesize that compared to those who feel less autonomy, the people who believe they are autonomous and have control over their lives also believe that (1) income inequality is lower and (2) income inequality is more acceptable. Using a representative sample of 3,427 Americans, we find evidence to support these hypotheses.

## Introduction

In the American dream, freedom comes with opportunities for prosperity if people work hard, make sacrifices, and take risks. Success is not limited by social class. This ideal may explain Americans’ tolerance for greater income inequality relative to the tolerance of citizens from other wealthy countries. How much income inequality is there in the US? Most economists argue that it has risen since the 1960s [1, 2]. As a result, researchers have sought to understand the implications of increased income inequality as it affects economic growth [3], attitudes toward redistributive policies [4–6], and well-being [7–9].

Numerous studies have found that perceptions of inequality in the US are systematically biased [10, 11]. In particular, Americans tend to underestimate the extent of both wealth inequality [12] and income inequality [13]. Moreover, Americans overestimate prospects of intergenerational mobility [14] (although see [15]). Estimates of inequality and mobility, in turn, affect policy preferences in some settings [16]. However, providing information about the actual income distribution in the US has small effects on redistributive policy preferences [6]. Moreover, experiencing greater inequality in one’s life increases tolerance for inequality [13, 17]. In short, the impact of the perceptions concerning the socio-economic environment on political preferences is mixed.

Misperceptions of inequality fall within a broader category of misperceptions of economic and political facts [18]. Reasons for these misperceptions include overreliance on one’s local context when drawing general conclusions [19], ideologically motivated reasoning [20, 21], and lack of numerical skills [22–24]. In this paper, we propose a novel mechanism for perceptions of inequality and the evaluation of its fairness. We posit that the more control people feel they have over their lives—their perceived *autonomy*—the less income inequality they will perceive (a negative correlation) and the more fair they will judge any inequality they perceive (a positive correlation).

Previous research has shown a connection between an internal locus of control and belief in a just world [25], as well as a connection between a stronger belief in free will and greater acceptance of income inequality [26]. Likewise, being primed with the general idea of choice (either one’s own or other people’s choices) increases acceptance of wealth inequality [27]. Moreover, researchers have found that belief in a just world is correlated both with a greater acceptance of income inequality and a greater tolerance of inequality [13].

A large literature in economics provides ample evidence that beliefs about fairness of the economic environment and views about meritocracy are related to tolerance for inequality. For instance, experimental studies show that having ex-ante *fair* chances to win a lottery decreases participants’ willingness to redistribute unequal ex-post outcomes [28–31]. Likewise, several studies have found that people are more accepting of inequality when income differences are due to achievements or choices rather than luck [32–36]. In general, people with meritocratic views of fairness are more tolerant of income inequality than those with egalitarian views of fairness [33].

Relatively little is known about how psychological factors are linked to perceptions of the socio-economic environment. In this paper we address the question of how beliefs about personal autonomy relate to perceptions of income inequality. We hypothesize that individuals with a greater sense of autonomy perceive the world as having less inequality and judge the inequality that exists to be acceptable. Highly autonomous people believe that inequality is largely due to differences in ability and effort. It is possible that those who think inequality is primarily due to differences in effort and ability also perceive less inequality than actually exists. Recognizing that inequality may be caused by situational variables that are beyond one’s control would clash with the view that people are autonomous and masters of their fates. If, as we argue below, individuals who perceive themselves as autonomous are more likely to think that inequality results from poor choices, these individuals might also believe that, since we all have the opportunity to use our capabilities to the best, fewer people, and especially fewer deserving people, are left behind. Our main hypothesis is that a *greater sense of autonomy is correlated with perceptions of less income inequality*. To test this hypothesis, we conducted a representative survey of Americans in which we asked respondents a series of questions about their perceptions of inequality in the US, their beliefs about the fairness of that inequality, and their perceived sense of autonomy.

To foreshadow our results, and in line with prior research [26], we find that greater perceived autonomy is correlated with beliefs that inequality is fair. Consistent with our main hypothesis, we also find that higher levels of perceived autonomy are correlated with the perception that income inequality in the US is lower than the data indicate. Moreover, we demonstrate that perceptions of fairness mediate the relationship between perceived autonomy and perceived income inequality. These findings uncover a novel relationship between perceptions of inequality and personal autonomy, and offer a new explanation for the observed misperceptions of income inequality. Furthermore, we find that higher perceived autonomy correlates with the belief that differences in income are largely due to effort and ability rather than luck.

### Psychological theories of autonomy

We measure perceptions of autonomy using established psychological scales. In particular, our main measure is Deci and Ryan’s scale of autonomy. Deci and Ryan proposed one of the most influential accounts of autonomy, self-determination theory (SDT). The basic tenet is that human beings have a fundamental need to be autonomous [37, 38], where autonomy is defined as regulation by the self, i.e., a capacity for and desire to experience self-regulation. Autonomous behavior is thus perceived as self-endorsed. Hence, people with a higher sense of autonomy believe that they are in greater control of their behavior and that external factors exert less influence [39]. Research shows that when people are made to feel more autonomous, they report higher levels of self-esteem, perceived competence, and well-being [37, 40]. In order to capture these basic concepts of self-determination theory, we use the General Index of Autonomy included in the Basic Personality Needs scale by Deci & Ryan [41].

The concept of autonomy is related, but distinct from, self–efficacy, which denotes an individual's belief in her ability to achieve goals and meet situational demands [42, 43]. It is a personal judgment of how well one can perform the actions required to handle a given situation. Expectations of one’s own self-efficacy determine whether an individual can cope with challenges and persist in the face of obstacles. Thus, individuals with greater self-efficacy will exert more effort and persevere. According to Bandura, self-efficacy is domain-specific [42, 43]. In his view, the efficacy belief system is not a global trait, but instead a differentiated set of self-beliefs linked to distinct domains of functioning. Others have conceptualized ‘generalized self-efficacy’ as a broad and stable sense of personal competence. In this view, general self-efficacy (GSE) is a basic belief in one’s competence to cope with a broad range of stressful or challenging demands [44, 45]. Across various domains, GSE is positively correlated with measures of domain-specific self-efficacy including exercise, abstinence from smoking [44], career-decision making, and math [46], among others. Therefore, in our survey, we use a measure of GSE to show the robustness of our findings to the use of different autonomy-related measures [45].

## Materials and methods

The study was approved by the University of Pennsylvania IRB under protocol number 830034. Consent was obtained in written form before the beginning of the survey. We used Qualtrics Online Panels to conduct our survey, which was fielded during June and July 2019. The study was pre-registered on OSF on June 24, 2019. The sample consists of 3,427 American adults living within the 50 states, the District of Columbia, and Puerto Rico. Based on 2017 estimates from the Census Bureau, the sample had intended quotas for different demographic groups in order to ensure representativeness. The target Census-based quotas and the composition of the sample are described in Table 1. The average age of respondents was 46.6 years (s. d. 17.6), and the median range of annual household income in 2018 was $50,000-$55,000. According to the US Census Bureau’s American Community Survey, the actual median household income was approximately $63,000 in 2018 [47].

**Table 1:** Sample demographic characteristics.

Variable	Quota (%)	Sample (%)
Age		
18–30	30.0	26.0
31–54	36.0	38.3
55+	34.0	35.7
***Race***		
White	61.9	60.2
Black	12.3	15.6
Hispanic	17.4	16.7
Other	8.4	7.5
***Gender***		
Male	49.0	44.9
Female	51.0	55.1
***Region***		
South	37.0	36.9
Midwest	22.0	22.2
Northeast	18.0	18.1
West	23.0	22.8
***Income***		
Less than $25k	18.0	18.3
$25-45k	22.0	22.4
$45-135k	46.0	47.6
$135k+	14.0	11.7

The survey consists of four modules. The first three modules focus on income, education and healthcare respectively. We measure respondents’ perceptions of domain-specific inequality, domain-specific autonomy and fairness in each module. The fourth module contains questions about domain-independent autonomy and well-being.

### Autonomy perceptions

We measure perceptions of autonomy using the General Index of Autonomy, which measures the average agreement on a scale from 1 to 7 with seven statements (e.g. “I feel like I am free to decide for myself how to live my life”) taken from the Basic Personality Needs Scale [39]. This scale includes reverse coding the questions that were posed in the negative (Cronbach’s alpha 0.81). In the supporting information, we also use the measure of generalized self-efficacy developed by Schwarzer and Jerusalem as a robustness check [43]. It assesses respondent’s agreement with ten statements on a scale from 1 to 7 (e.g., “I am certain that I can accomplish my goals”) (Cronbach’s alpha is 0.94). We include summary statistics for both scales in the S1 File.

### Perceptions of inequality

The survey measures perceptions of income inequality by asking what proportion of *households* in the US were in three annual pre-tax income categories: less than $45,000, between $45,000 and $135,000, and more than $135,000. These income categories roughly define a household of three as lower, middle class, and upper class [48]. Lower perceived inequality is indicated by a lower estimated proportion of households with income below $45,000, a higher estimated proportion of households with an income between $45,000 and $135,000, and a higher estimated proportion with an income greater than $135,000. Importantly, these questions elicit beliefs about the distribution of income across brackets, one aspect of inequality. However, they do not capture another aspect of inequality, namely beliefs about the concentration of income. In other words, our measure cannot capture beliefs about inequality such as perceptions of extremely high incomes by top income earners. Even though this variable may paint an incomplete picture of a person’s overall perception of inequality, we believe that it provides a meaningful measure of how unequal a person perceives total income distribution within the US. Complementing the numeric question, we also ask respondents in a yes or no question to state whether they believe large household income differences exist among Americans. Additionally, we ask respondents to state their beliefs about the proportion of people in each of the income groups who do not have a college education, as well as the proportion of people who do not have health insurance. A lower stated proportion in the poorest group and a higher proportion in the richest groups indicates lower perceptions of inequality in the domains of education and health care. We report results for college education and health insurance in the S1 File.

### Fairness perceptions

Perceptions of fairness are measured with ten questions describing various situations (e.g. “How fair is it that some Americans have billions of dollars while others have very little?” and “Is it fair that Americans with higher incomes can buy better healthcare for themselves and their children while others get much worse healthcare?”) which were evaluated on a continuous scale from “Very Unfair” to “Very Fair”. Respondents use a slider ranging from -1 to 1 and we recode these answers to range from 0 (Very Unfair) to 1 (Very Fair). (Cronbach’s alpha across the ten questions is 0.87). Because no standard scale is available for measuring perceived fairness, we conduct a principal component analysis on the questions. The first principal component represents perceived fairness of inequality in general (mean = 0, s.d. = 2.29, min = -3.71, max = 7.04). Higher values convey greater perceived fairness. In the supporting information, we summarize our measures of fairness.

### Merit perceptions

In the survey, we ask three questions about people’s beliefs about the extent to which hard work, ability, and luck determine differences in life outcomes among Americans. Each question is answered on a scale from ‘Not at all’ (0) to ‘Completely’ (100). In particular, respondents are asked: “How much do you think the differences in income among Americans can be explained by hard work?” (mean = 50.41, s.d. = 27.51), “How much do you think the differences in income among Americans can be explained by talents and abilities?” (mean = 53.96, s.d. = 25.78), and “How much do you think the differences in income among Americans can be explained by luck?” (mean = 42.83, s.d. = 26.94). Respondents also answer the general question “Do you agree with the following statement? ‘In the US everybody has a chance to make it and be successful’”, indicating their answer on a scale from ‘Strongly disagree’ (0) to ‘Strongly agree’ (1) (mean = 0.55, s.d. = 0.30).

### Control variables

We also measure demographic variables, which we include as controls in our analyses. These include age, education (in five categories from less than high school to more than 4-year college), annual household income category (in $5,000 increments from less than $5,000 to more than $135,000), partisan identification (Democrat, Republican, Independent or Other), racial identification (white, black, Hispanic or Latino, or other), state of residence, and gender identification. As a proxy for experienced income mobility, we include variables measuring a respondent’s perceived income relative to the average income in the US, both now and growing up (in 0 to 100 scale, where 0 is ‘Much less’ and 100 ‘Much more’).

Generally, we conduct inferential analyses using continuous variables, although we sometimes describe results using a median split of the General Index of Autonomy (e.g., Tables 2 and 6).

**Table 2:** Average perceived and *true* proportion size in each income group.

	Less than $45,000	Between $45,000 and $135,000	More than $135,000
(a) Avg. High Autonomy	41.23	39.49	19.28
(b) Avg. Low Autonomy	44.76	37.65	17.58
(c) Census Bureau 2018	45.52	39.45	15.03

Notes: The numbers from the Census Bureau are calculated for the entire population in 2018 using the American Community Survey Public Use Microdata Sample and housing weights.

## Results

### Autonomy and perceptions of inequality

Before discussing relationships among concepts, we analyze perceptions of inequality separately to gain a better understanding of the data. Table 2 illustrates differences in perceptions of income inequality between those with high and low levels of own autonomy, as defined by a median split of the General Index of Autonomy. Average responses are shown for the perceived income inequality questions for high and low autonomy groups (demographic characteristics of these groups are presented in the S1 File). Table 2 also compares these averages with true proportions from the US Census Bureau’s American Community Survey in 2018. The table indicates that people with high versus low autonomy perceive different levels of inequality (for each of the three income levels, the t-test of differences in means is significantly different from zero at p < .01).

Table 2 also shows that less autonomous people are more *accurate* in their estimates of the percentage of households in the lowest income group with an income below $45,000 (p-value of the t-test of the difference from the true level is 0.09). People who perceive themselves as more autonomous overestimate the proportion of people in the highest income group and underestimate the proportion in the lowest income group (the p-values of the t-tests of differences with the true size are less than 0.01). Thus, a systematic tendency exists among high autonomy individuals to estimate fewer poor people. Compared to the lower autonomy group, the higher autonomy group estimates 3.5% fewer people in the ‘less than $45,000’ group. S7 and S8 Tables in S1 File indicate estimations of the shares of people in each income group without health insurance and without a college degree. Regardless of their autonomy level, people underestimate the proportion of individuals without a college degree and overestimate the proportion without health insurance. However, overall, the tables also show that across domains, people with lower autonomy generally perceive greater inequality than those with higher inequality. In particular, individuals with low autonomy estimate higher proportions of people at the lower end of the income spectrum and lower proportions of people at the higher end of the income spectrum than those with higher autonomy.

To control for demographic variables, we estimate versions of the following linear regression through ordinary least squares (OLS):

$$Y_{i}=\beta_{0}+\beta_{0}A_{i}+\gamma X_{i}+\in_{i}$$ (1)

where *Y* is the estimate of the size of the income groups (those who earn less than $45,000, between $45,000 and $135,000, and more than $135,000); *A* is the measure of autonomy; *X* is a vector of demographic covariates including: age, partisan identification, gender, race, income, perceived income relative to average now, perceived income relative to average growing up, and educational level; and ϵ is the error term. Control variables that are not continuous are included as categorical variables, using an indicator for each category. Table 3 presents the estimated values of β_1_ for separate regressions, in which the dependent variable is the perceived percent of households that earn less than $45,000, between $45,000-$135,000 and more than $135,000. It shows that higher levels of perceived autonomy are correlated with lower estimates of the percentage of households in the poorest group (less than $45,000), as well as higher estimates of the size of the middle (between $45,000 and $135,000) group. Thus, we show that the above result is robust to controlling for various demographic variables, experienced (income) mobility and perceived relative income.

**Table 3:** Results of OLS regressions of perceived income inequality on autonomy.

		Dependent Variable:			
	Less than $45,000	Between $45,000 and $135,000	More than $135,000	Difference between Less than $45,000 and more than $135,000	Are there large income differences?
General Index of Autonomy	-1.014**	.654*	.361	-1.375**	-.010**
	(.318)	(.274)	(.222)	(.476)	(.004)
DV Average	43.03	38.55	18.41	24.62	0.96
N	3427	3427	3427	3427	3427
R^2^	.11	.13	.07	0.08	0.03

Notes: *p<0.05,

**p<0.01,

***p<0.001. Each column shows the unstandardized coefficient of the General Index of Autonomy in an OLS regression in which the variable at the top of the column is the dependent variable when including controls. Robust standard errors are in parentheses. Controls for every column include age, perceived current and past economic income relative to average, and dummies for gender, race, state, income group, education level, and party ID. The range for the General Index of Autonomy is 1.14 to 7, with higher values indicating greater autonomy. As a reference, the table also reports the average value of the dependent variable.

Table 3 furthermore shows that a one-point increase in the General Index of Autonomy (which empirically in our sample goes from 1.14 to 7) is related to a decrease of 1.014 percentage points in the estimated proportion of households earning less than $45,000 a year. A one-point increase in the General Index of Autonomy is also correlated with an increase of 0.654 percentage points in the estimated percentage of households earning $45,000 to $135,000. Controlling for a host of variables, individuals with higher levels of General Autonomy estimate that there are fewer poor and more middle class households. The results also show a slightly higher estimation of the proportion of households earning more than $135,000 a year, though this estimate is not statistically significantly different from zero. However, the difference between the estimated proportion of households earning less than $45,000 and more than $135,000 shrinks as perceived autonomy increases. A one-point increase in the General Index of Autonomy is related to a 1.375 decrease in the difference. Finally, Table 3 shows that a one-point increase in the General Index of Autonomy is correlated with a one-percentage point decrease in the probability of responding yes to the question “Are there large household income differences among Americans?” Going from the lowest value of the General Index of Autonomy to the highest would translate to a 7 percent difference in the probability of saying “yes” when responding to the question. Since, overall, 96 percent of our sample answered the question affirmatively, this is a meaningful result. Hence, the General Index of Autonomy is correlated with perceiving less inequality, lending support to our main hypothesis. As S9 to S11 Tables in S1 File show, results are similar, though more muted, when using the General Self-Efficacy Index as the main independent variable and when using measures of inequality in the domains of attainment in higher education and access to healthcare as the dependent variables.

As we show in the next section, if individuals with high perceived autonomy believe society is basically fair and meritocratic, they will tend to believe that low income people deserve to be poor, attributing poverty to lower effort and ability rather than lack of access to educational or health services.

### How autonomous is everyone else?

We hypothesized that individuals with higher perceived autonomy perceive less inequality and believe the existing inequality to be fair. Presumably, to conceive inequality as fair, one is likely to believe that success is based on effort and is within one’s control. Furthermore, in order to maintain this belief, it would be reasonable to posit that one’s own perceived autonomy generalizes to others. In this view, people are likely to believe that one’s own level of control over life circumstances and outcomes is similar to that of others. Whether this is the case is an empirical question. For example, a person might perceive her own autonomy and freedom to be high, but acknowledge the fact that others might have less autonomy and, thus, fewer chances to be successful. Therefore, they would discount the role of merit and the overall fairness of the economic system.

Past research has found that a belief in free will, or the ability to act and choose freely, is positively correlated with measures of perceived personal autonomy. For example, Paulhus and Carey [49] found a positive correlation between the belief that people in general have free will, and the belief in a higher own internal locus of control, as measured by the Multidimensional Locus of Control Inventory (see also [50]).

**Fig 1:**
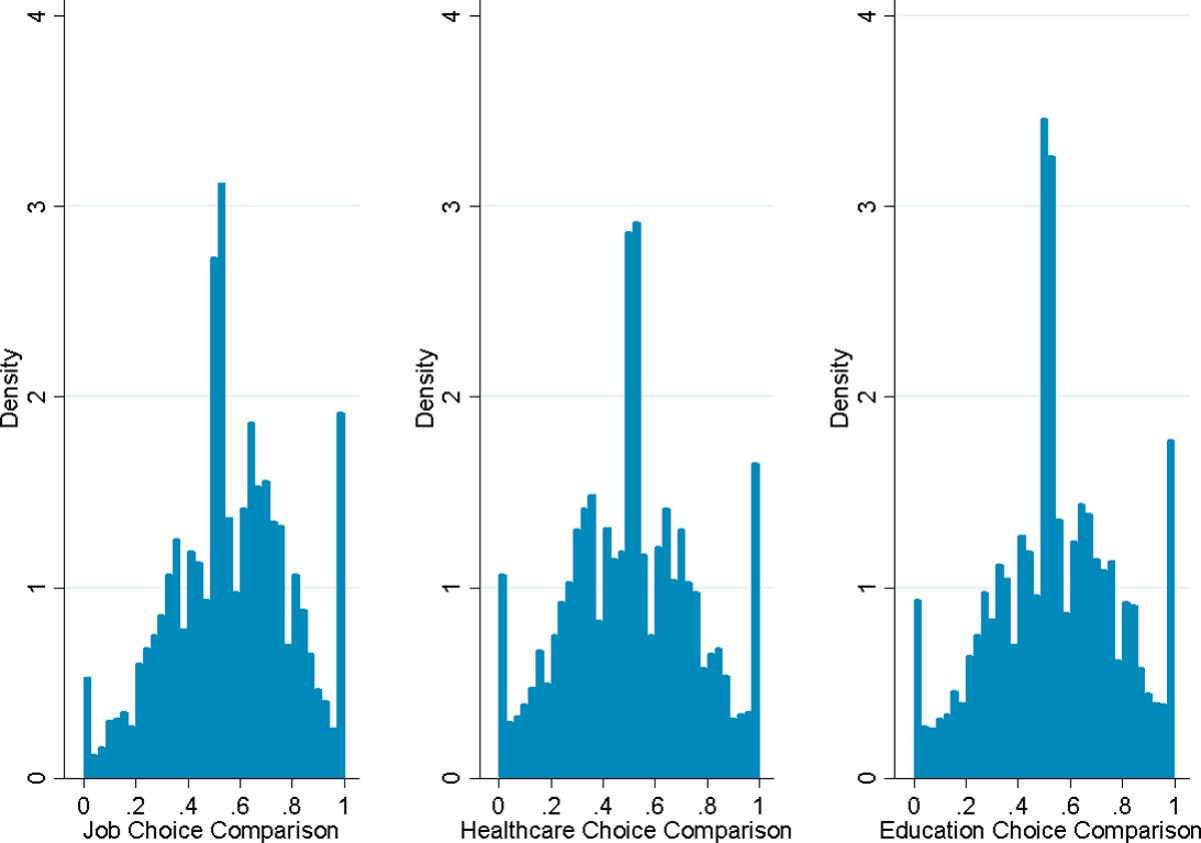
Distributions of comparison of choices of others to own.

Our survey allows us to test the hypothesis that personal perceived autonomy generalizes to the perceptions of others. For each of three categories (income, education and healthcare), we ask respondents if they believe they have more, less or about the same amount of free choice as ‘typical Americans’. More specifically, we ask people if they believe others have more or fewer choices than they do when it comes to finding and keeping a desirable job, selecting healthcare providers, and selecting schools for themselves or their children. Each question is answered on a continuous scale from 0 (‘Many fewer’) to 1 (‘Many more’). As shown in Fig 1, answers are clustered around the middle of the scale, suggesting that many people believe that others have similar choice sets to their own. These results do not depend on the level of personal autonomy, as shown in S6 Table of S1 File. The only exception is the case of education, in which an increase in autonomy is correlated with the perception that there are more people who have more choices than oneself when it comes to selecting schools.

Our findings could be explained by the fact that individuals tend to overestimate the degree to which their beliefs, preferences, or behavior are typical of others [51, 52]. In sum, our results suggest that people, regardless of their perceived autonomy, tend to believe that others have about the same degree of choices in their lives as they do. This is important to consider in order to fully understand how perceived personal autonomy relates to perceptions of inequality and fairness.

The finding that people believe that others have choices in their lives similar to theirs is also supported by the fact that people with higher levels of perceived autonomy are more likely to agree with the following statement: “In the US everybody has a chance to make it and be successful.” As Table 4 shows, a one-point increase in the General Autonomy Index is related to a 0.054 increase, on a 0 to 1 scale, in agreement with the statement.

**Table 4:** Results of OLS regression of belief in opportunity on autonomy.

	Dependent Variable:
	In the US, everybody has a chance to make it and be successful
General Index of Autonomy	.054***
	(.005)
N	3427
R-squared	.23

Notes: *p<0.05,

**p<0.01,

***p<0.001. Each column shows the unstandardized coefficient of the General Index of Autonomy in an OLS regression in which the variable at the top of the column is the dependent variable when including controls. Robust standard errors are in parentheses. Controls for every column include age, perceived current and past economic income relative to average, and dummies for gender, race, state, income group, education level, and party ID. The range for the General Index of Autonomy is 1.14 to 7, with higher values indicating greater autonomy.

### Autonomy and fairness

In this section, we look at the relationship between autonomy and perceived fairness. The following table presents regressions predicting fairness (how fair are economic, educational, and healthcare results) from autonomy, controlling for demographic variables. The estimated regressions are similar to Eq (1), but with fairness as the dependent variable. Table 5 shows that all relationships are positive and statistically significant, with a unit increase in the General Index of Autonomy corresponding to an increase of 0.210 points in our measure of overall fairness, ceteris paribus.

**Table 5:** Results of OLS regression of perceived fairness on autonomy.

	Dependent Variable:				
	Overall fairness	Is the US economic system fair?	Is it fair for there to be billionaires and others with very little?	Fair rich have access to better healthcare	Fair rich have access to better education
General Index of Autonomy	.210***	.041***	.023***	.019***	.016**
	(.034)	(.005)	(.005)	(.005)	(.005)
N	3427	3427	3427	3427	3427
R^2^	.32	.29	.20	.17	.16

Notes: *p<0.05,

**p<0.01,

***p<0.001. Each column shows the unstandardized coefficient of the General Index of Autonomy in an OLS regression in which the variable at the top of the column is the dependent variable when including controls. Robust standard errors are in parentheses. Controls for every column include age, perceived current and past economic income relative to average and dummies for gender, race, state, income group, education level, and party ID. The range for the General Index of Autonomy is 1.14 to 7, with higher values indicating greater autonomy. The range for the Overall fairness measure is -3.71 to 7.04 with higher values indicating greater perceived fairness.

Additionally, Table 5 shows the correlations between own autonomy and some of the questions that comprise our overall fairness measure, all of which are answered on a continuous scale from 0 to 1. An increase of one point in the General Index of Autonomy measure is associated with a 0.041 increase in the response to the question “Is the US economic system fair?” Similarly, a one-point increase in autonomy is associated with a 0.023 increase in the response to the question of whether the existence of billionaires is fair. Autonomy is also correlated with an increase of 0.019 and 0.016 points in answers to questions about whether it is fair that richer people have better access to healthcare and education, respectively. Thus, across various measures, perceived personal autonomy is positively correlated with the belief that one’s economic and social environment is basically fair.

In the previous section, we showed that some participants believe their levels of autonomy apply to others. Yet, our data also indicate that a substantial fraction of participants do not make that assumption and recognize that others differ. We therefore test the extent to which the relationship between perceived autonomy and fairness depends on the belief that others have a similar level of autonomy. Table 6 presents regressions of fairness on perceived autonomy, including an interaction between perceived personal autonomy and a variable indicating whether others are perceived to have more or fewer options when choosing a job, a school, or a healthcare provider. For ease of interpretation, we dichotomize both variables. For autonomy, we divide participants into those with high and low autonomy according to a median split as before; for choices, we divide participants into those who perceive others to have the same or more choices as themselves, and those who perceive others to have fewer choices. The results from all three columns of Table 6 show a positive correlation between personal autonomy and the extent to which a person perceives society to be fair; however, this correlation is only significant for those who believe others have *at least as many choices* as they have. The power of perceived personal autonomy to predict a person’s views about fairness is stronger if one believes that others have as much or more freedom of choice as they themselves enjoy.

**Table 6:** Results of OLS regression of perceptions of fairness on autonomy by perceptions of others choice in the domains of income, education, and healthcare.

	Dependent Variable:		
	Fairness		
	(1)	(2)	(3)
High Autonomy	.226	.192	.257*
	(.118)	(.102)	(.114)
Others Have More Income Choices	.551***		
	(.092)		
High Autonomy × Others Have More Income Choices	.085		
	(.139)		
Others Have More Healthcare Choices		.419***	
		(.090)	
High Autonomy × Others Have More Healthcare Choices		.185	
		(.133)	
Others Have More School Choices			.508***
			(.094)
High Autonomy × Others Have More School Choices			.182
			(.139)
Marginal Effects of High Autonomy (others more choices = 0)	.226	.192	.257*
	(.118)	(.102)	(.114)
Marginal Effects of High Autonomy (others more choices = 1)	.311***	.376***	.439***
	(.087)	(.095)	(.089)
N	3427	3427	3427
R^2^	.33	.33	.31

Notes: *p<0.05,

**p<0.01,

***p<0.001. Every column reports the unstandardized OLS regression coefficient for the High Autonomy (1 if yes, 0 if no), as defined by a median split of the General Index of Autonomy) and whether the respondent believes others have more choices than themselves (1 if yes and 0 if no) in a regression in which our measure of fairness is the dependent variable including controls. Robust standard errors are in parentheses. Controls for every column include age, perceived current and past economic status, and dummies for gender, race, state, income group, education level, and party ID. The range for the Overall fairness measure is -3.71 to 7.04 with higher values indicating greater perceived fairness.

Overall, we conclude that the greater one’s perceived own autonomy, the more likely one is to perceive the economic system as fair. To better understand the underlying reasons, we now turn to analyzing the perceived causes of inequalities.

### Autonomy and the causes of inequality

We now examine the relationships among autonomy and beliefs about the role of hard work, ability, and luck in determining differences in income. We ask respondents three questions about the extent to which they believe hard work, ability, or luck each determines differences in life outcomes (on a slider scale from 0 to 100). We estimated regressions with these responses as the dependent variable and perceived own autonomy as the main independent variable. Results are presented in Table 7. Higher levels of autonomy are significantly correlated with the belief that differences in income are due to hard work and ability rather than luck. A one-unit increase in the General Index of Autonomy is correlated, ceteris paribus, with a 3.772 point increase in the belief that differences in income are due to hard work, a 4.318 increase points in the belief that they are caused by differences in ability, and a 2.818 decrease in the belief that they are caused by luck.

**Table 7:** Results of OLS regression of perceived causes of differences in income on autonomy.

	Dependent Variable:		
	Differences in Income due to Hard Work	Differences in Income due to Ability	Differences in Income due to Luck
General Index of Autonomy	3.772***	4.318***	-2.818***
	(.481)	(.455)	(.497)
N	3427	3427	3427
R^2^	.13	.14	.07

Notes: *p<0.05,

**p<0.01,

***p<0.001. Each column shows the unstandardized coefficient of the General Index of Autonomy in an OLS regression in which the variable at the top of the column is the dependent variable when including controls. Robust standard errors are in parentheses. Controls for every column include age, perceived current and past economic status, and dummies for gender, race, state, income group, education level, and party ID. The range for the General Index of Autonomy is 1.14 to 7, with higher values indicating greater autonomy.

The results in Table 7 show that people with greater perceived personal autonomy are more likely to say that income differences in the US are due to hard work and ability rather than luck. This finding resonates with our previous result that individuals with high levels of perceived autonomy believe that everyone in the US has an equal opportunity to be successful (Table 4).

Taken together, these results mean that perceiving oneself as highly autonomous is associated with a belief in a meritocratic worldview and the existence of equal opportunities. These individuals are more tolerant of income inequality because they think people tend to get the economic outcomes they deserve.

### Fairness as a mediator

**Fig 2:**
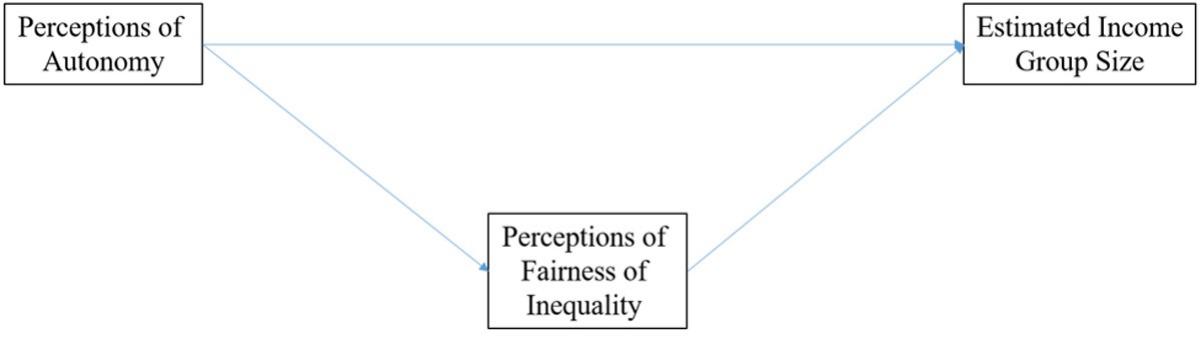
Relationship between estimated income group size and autonomy mediated by perceptions of fairness.

Our results have underscored the fact that greater perceived personal autonomy is positively correlated both with lower levels of perceived income inequality and higher levels of perceived fairness. We now investigate whether views about fairness *mediate* the relationship between autonomy and inequality. In other words, we decompose the association in a *direct link* and an *indirect link* via individuals’ fairness views, as depicted in Fig 2. Table 8 displays the results for income inequality as measured by the estimated sizes of different income groups.

**Table 8:** Fairness as a mediator of the relationship between perceived autonomy and estimated sizes of income groups (Inequality).

Dependent Variable	Direct Effect of Autonomy	Indirect Effect of Autonomy (through Fairness)	Total Effect
Perceived % Income < $45,000	-0.69*	-.32***	-1.01**
	(0.31)	(0.06)	(0.31)
Perceived % Income $45,000-$135,000	0.42	.23***	0.65*
	(0.27)	(0.05)	(0.27)
Perceived % Income > $135,000	0.27	0.09***	0.36
	(0.22)	(0.03)	(0.22)

Notes: *p<0.05,

**p<0.01,

***p<0.001. Each row presents the direct effect and the effect mediated by fairness of the General Index of Autonomy. Robust standard errors are in parentheses. Controls for every column include age, perceived current and past economic status, and dummies for gender, race, state, income group, education level, and party ID.

The coefficients in Table 8 show that the indirect link via fairness views is an important driver of the relationship between perceived autonomy and inequality. It is statistically significant and captures roughly one third of the effect size in each estimation in Table 8. The direct effect, on the other hand, is not statistically significant though it explains a large portion of the total effect size, with the exception of the first row in which the size of the poorest income group is the dependent variable. We therefore conclude that the association between perceptions of autonomy and inequality is at least partly mediated by views of fairness. Thus, the correlation between perceiving greater personal autonomy and perceiving less inequality can partly be explained by the fact that people with high autonomy tend to hold the view that the current income distribution is fair.

## Discussion

We find support for our primary hypothesis that those who perceive themselves as highly autonomous tend to perceive less income inequality in the US. Our results also show that people who perceive themselves as more autonomous believe the social and economic system in the US is generally fair, and that income differences stem from differences in effort and ability rather than luck. When individuals perceive themselves and others as being in control of their life outcomes, they are more likely to believe that people get what they deserve and deserve what they get. That is, they see the world as fair.

Our results are relevant to the current economic climate, with rising inequality in the US [1]. Studies based on tax data show an increase in income inequality, and further suggest that a substantial part of this inequality might be driven by factors outside an individual’s control (i.e., capital gains rather than hours worked or education attained) [2, 53]. We believe these perceptions have consequences, and they are an important topic for future studies.

One psychological explanation for our findings is that people may engage in motivated reasoning [54] to reduce cognitive dissonance and maintain a consistent world-view. More precisely, individuals who think that inequality is rather low and generally fair may be motivated to perceive themselves as in control of their lives and, importantly, avoid information that may disprove their belief. In the same way, those with high levels of perceived autonomy may search for and pay attention to information about (the sources of) inequality that is consistent with their self-perception.

Motivated reasoning in the second example can result in an accurate estimate of inequality, combined with the belief that a large proportion of the population does not work hard enough. Alternatively, motivated reasoning may lead one to, inaccurately, perceive less inequality, while retaining the belief that inequality is indeed caused by differences in talent and merit. Comparing subjective estimates of the distribution of income with actual data suggests that individuals with higher perceived autonomy tend to be more biased, as they underestimate the amount of existing inequality. We conjecture that, in order to maintain a coherent belief system, individuals with higher perceived autonomy might distort their perception to align it with their “belief in a just world” [55], i.e., the belief that the society they live in is basically fair and that individuals can choose the way of life they prefer. The belief that income inequality is relatively low can be maintained if one refrains from seeking out information about the actual degree of inequality that may disprove one’s belief. We have some initial evidence supporting this explanation.

Our study opens new avenues of inquiry. First, our analyses are purely correlational. Future studies should examine potential causal relationships. Perceived personal autonomy might be a part of a person’s identity that shapes her perceptions of inequality; establishing causality would require manipulating people’s perceptions of autonomy, which is a challenging task. On the other hand, perceptions of one’s autonomy could be caused by exposure to, or beliefs about, inequality. Future experiments may give us the answers. Finally, our study is limited to the context of our sample in the US, and we do not claim that it generalizes to populations outside of the US. The relationships we uncover may be driven by institutional and cultural factors that are unique to the American context.

To conclude, we hope this paper stimulates research that explores the impact of other psychological traits besides autonomy on perceptions of the economic environment, especially income inequality.

## Supporting information

S1 File (DOCX)

## References

[1] Piketty T. and Saez E., "Income inequality in the United States, 1913–1998," Q J Econ, vol. 118, no. 1, pp. 1–41, 2003.

[2] Piketty T., Saez E. and Zucman G., "Distributional national accounts: methods and estimates for the United States.," Q J Econ, vol. 133, no. 2, pp. 553–609, 2017.

[3] Alesina A. and Rodrik D., "Distributive politics and economic growth," Q J Econ, vol. 89, no. 5–6, pp. 465–490, 1994.

[4] Alesina A. and La Ferrara E., "Preferences for redistribution in the land of opportunities," J Public Econ, vol. 89, no. 5–6, pp. 897–931, 2005.

[5] Bartels L., "Homer gets a tax cut: Inequality and public policy in the American mind," Perspectives on Politics, vol. 3, no. 1, pp. 15–31, 2005.

[6] Kuziemko I., Norton M. I., Saez E. and Stantcheva S., "How elastic are preferences for redistribution? Evidence from randomized survey experiments," Am Econ Rev, vol. 105, no. 4, pp. 1478–1508, 2015.

[7] Alesina A., Di Tella R. and MacCulloch R., "Happiness and inequality: Are Europeans and Americans different?," J Public Econ, vol. 88, no. 9–10, pp. 2009–2042, 2004.

[8] Napier J. L. and Jost J. T., "Why are conservatives happier than liberals?," Psychol Sci, vol. 19, no. 6, pp. 565–572, 2008 10.1111/j.1467-9280.2008.02124.x 18578846

[9] Rivenbark J, Arseneault L, Caspi A, Danese A, Fisher HL, Moffitt TE, et al “Adolescents’ perceptions of family social status correlate with health and life chances: A twin difference longitudinal cohort study.” P NATL ACAD SCI USA. 2020 10.1073/pnas.1820845116 31907315 PMC7519389

[10] Hauser O. P. and Norton M. I., "(Mis) perceptions of inequality," Curr Opin Psychol, vol. 18, pp. 21–25, 2017 10.1016/j.copsyc.2017.07.024 29221507

[11] Gimpelson V. and Treisman D., "Misperceiving inequality," Economics & Politics, vol. 30, no. 1, pp. 27–54, 2018.

[12] Norton M. I. and Ariely D., "Building a better America—One wealth quintile at a time," Perspect Psychol Sci, vol. 6, no. 1, pp. 9–12, 2011 10.1177/1745691610393524 26162108

[13] Trump K.-S., "Income inequality influences perceptions of legitimate income differences," Br J Polit Sci, vol. 48, no. 4, pp. 929–952, 2018.

[14] Alesina A., Stantcheva S. and Teso E., "Intergenerational Mobility and preferences for redistribution," Am Econ Rev, vol. 108, no. 2, pp. 521–554, 2018.

[15] Cheng S, Wen F. “Americans overestimate the intergenerational persistence in income ranks.” P NATL ACAD SCI USA, 2019 7 9;116(28):13909–14. 10.1073/pnas.1814688116 31235566 PMC6628797

[16] Cruces G., Perez-Truglia R. and Tetaz M., "Biased perceptions of income distribution and preferences for redistribution: Evidence from a survey experiment.," J Public Econ, vol. 98, pp. 100–112, 2013.

[17] Roth C. and Wohlfart J., "Experienced inequality and preferences for redistribution," J Public Econ, vol. 67, pp. 251–262, 2018.

[18] Jerit J. and Zhao Y., "Political Misinformation," Annu Rev Polit Sci, vol. 23, pp. 77–94, 2020.

[19] Sands M. L., "Exposure to inequality affects support for redistribution," P Natl Acad Sci USA, vol. 114, no. 4, pp. 663–668, 2017 10.1073/pnas.1615010113 28069960 PMC5278453

[20] Kahan D.M., “Ideology, motivated reasoning, and cognitive reflection: An experimental study,” Judgm Decis Mak, vol. 8, no.4, pp 407–424, 2013.

[21] Bullock J. G., Gerber A. S., Hill S. J. and Huber G., "Partisan bias in factual beliefs about politics," Quart J Polit Sci, vol. 10, no. 4, pp. 519–578, 2015.

[22] Kahan D. M., Peters E., Dawson E., C. and Slovic P, "Motivated numeracy and enlightened self-government," Behav Public Policy, vol. 1, no. 1, pp. 54–86, 2017.

[23] Peters E., "Beyond comprehension: The role of numeracy in judgments and decisions," Curr Dir Psychol Sci, vol. 21, no. 1, pp. 31–35, 2012.

[24] Peters E., Innumeracy in the wild: Misunderstanding and misusing numbers, Oxford University Press, forthcoming.

[25] Lipkus I., "The construction and preliminary validation of a global belief in a just world scale and the exploratory analysis of the multidimensional belief in a just world scale," Pers Individ Dif, vol. 12, no. 11, pp. 1171–1178, 1991.

[26] Mercier B., Wiwad D., Piff P. K., Aknin L., Robinson A. R. and Shariff A., "Does Belief in Free Will Increase Support for Economic Inequality?," PsyArXiv, no. https://psyarxiv.com/k45ud/, 2018.

[27] Savani K. and Rattan A., "A choice mind-set increases the acceptance and maintenance of wealth inequality," Psychol Sci, vol. 23, no. 7, pp. 796–804, 2012 10.1177/0956797611434540 22700330

[28] Brock J. M., Lange A. and Ozbay E. Y., "Dictating the risk: Experimental evidence on giving in risky environments," Am Econ Rev, vol. 103, no. 1, pp. 415–437, 2013.

[29] Krawczyk M. and Le Lec F., ‴Give me a chance!’An experiment in social decision under risk," Exp Econ, vol. 13, no. 4, pp. 500–511, 2010.

[30] Cappelen A. W., Konow J., Sørensen E. Ø. and Tungodden B., "Just luck: An experimental study of risk-taking and fairness," Am Econ Rev, vol. 103, no. 4, pp. 1398–1413., 2013.

[31] Freundt J. and Lange A., "On the determinants of giving under risk," J Econ Behav Organ, vol. 142, pp. 24–31, 2017.

[32] Akbaş M., Ariely D. and Yuksel S., "When is inequality fair? An experiment on the effect of procedural justice and agency," J Econ Behav Organ, vol. 161, pp. 114–127., 2019.

[33] Almås I., Cappelen A. W. and Tungodden B., "Cutthroat Capitalism versus Cuddly Socialism: Are Americans More Meritocratic and Efficiency-Seeking than Scandinavians?" J Polit Econ, forthcoming.

[34] Cappelen A. W., Hole A. D., Sørensen E. Ø. and Tungodden B., "The pluralism of fairness ideals: An experimental approach," Am Econ Rev, vol. 97, no. 3, pp. 818–827, 2007.

[35] Cappelen A. W., Sørensen E. Ø. and Tungodden B., "Responsibility for what? Fairness and individual responsibility," Eur Econ Rev, vol. 54, no. 3, pp. 429–441, 2010.

[36] Piff P. K., Wiwad D., Robinson R., Aknin L. B., Mercier B. and Shariff A., "Shifting attributions for poverty motivates opposition to inequality and enhances egalitarianism," Nat Hum Behav, 2020 10.1038/s41562-020-0835-8 32203322

[37] Deci E. L. and Ryan R. M., "Motivation, personality, and development within embedded social contexts," in Oxford Handbook of Human Motivation, Oxford, Oxford University Press, 2012, pp. 85–107.

[38] Deci E. L. and Ryan R. M., "The general causality orientations scale: Self-determination in personality," J Res Pers, vol. 19, no. 2, pp. 109–134, 1985.

[39] Deci E. and Ryan R., "The support of autonomy and the control of behavior," J Pers Soc Psychol, vol. 53, no. 6, pp. 1024–1037, 1987 10.1037//0022-3514.53.6.1024 3320334

[40] Weinstein N., Przybylski A. K., & Ryan R. M. “The index of autonomous functioning: Development of a scale of human autonomy.” J Res Pers, 2012, 46(4), 397–413.

[41] Deci E. L. and Ryan R. M., "Basic Psychological Needs Scales," 2006 [Online]. Available: m http://www.psych.rochester.edu/SDT/.

[42] Bandura A., "Guide for constructing self-efficacy scales.," in Self-Efficacy Beliefs of Adolescents, vol. 5, Pajares F. and Urdan T., Eds., Information Age Publishing, 2006, pp. 307–337.

[43] Bandura A., "Perceived self-efficacy in cognitive development and functioning," Educational Psychologist, vol. 28, no. 2, pp. 117–148, 1993.

[44] Luszczynska A., Scholz U. and Schwarzer R., "The general self-efficacy scale: multicultural validation studies," J Psychol, vol. 139, no. 5, pp. 439–457, 2005 10.3200/JRLP.139.5.439-457 16285214

[45] Schwarzer R. and Jerusalem M., "Generalized self-efficacy scale," in Measures in health psychology: A user’s portfolio. Causal and control beliefs, vol. 12, Windsor, England, NFER-Nelson, 1995, pp. 35–37.

[46] Betz N. E. and Klein K. L., "Relationships among measures of career self-efficacy, generalized self-efficacy, and global self-esteem," J Career Assess, vol. 4, no. 3, pp. 285–298., 1996.

[47] Rothbaum J. and Edwards A. “U.S. Median Household Income was $63,179 in 2018, Not Significantly Different from 2017,” United States Census Bureau Available from: https://www.census.gov/library/stories/2019/09/us-median-household-income-not-significantly-different-from-2017.html [Accessed November 30, 2020].

[48] Kochhar R. “The American middle class is stable in size, but losing ground financially to upper-income families,” Pew Research Center Available from: https://www.pewresearch.org/fact-tank/2018/09/06/the-american-middle-class-is-stable-in-size-but-losing-ground-financially-to-upper-income-families/ [Accessed November 19, 2020].

[49] Paulhus D. L. and Carey J. M., "The FAD–Plus: Measuring lay beliefs regarding free will and related constructs," J Pers Assess, vol. 93, no. 1, pp. 96–104., 2011 10.1080/00223891.2010.528483 21184335

[50] Crescioni A. W., Baumeister R. F., Ainsworth S. E., Ent M. and Lambert N. M., "Subjective correlates and consequences of belief in free will.," Philos Psychol, vol. 29, no. 1, pp. 41–63, 2016.

[51] Ross L., Greene D. and House P., "The ‘false consensus effect’: An egocentric bias in social perception and attribution processes," J Exp Soc Psychol, vol. 13, no. 3, pp. 279–301, 1977.

[52] Marks G. and Miller N. "Ten years of research on the false-consensus effect: An empirical and theoretical review," Psychol Bull, vol. 102, no. 1, pp. 72–90, 1987.

[53] Hussey A. and Jetter M., "Long term trends in fair and unfair inequality in the United States," Appl Econ, vol. 49, no. 12, pp. 1147–1163, 2017.

[54] Kunda Z., "The Case for Motivated Reasoning," Psychol Bull, vol. 108, no. 3, pp. 480–498, 1990 10.1037/0033-2909.108.3.480 2270237

[55] Benabou R. and Tirole J., "Belief in a just world and redistributive politics," Q J Econ, vol. 121, no. 2, pp. 699–746, 2006.

