# Supplementary material for: How perceptions of autonomy relate to beliefs about inequality and fairness
Source: PLoS One. 2021 Jan 13;16(1):e0244387. doi: 10.1371/journal.pone.0244387 (PMC7806156; doi:10.1371/journal.pone.0244387)
Supplement: S1 File — (DOCX) [file pone.0244387.s001.docx]

## Autonomy and Fairness

In the following tables, we present descriptive statistics for our measures of autonomy, self-efficacy and fairness. We also show the results of the principal component analysis of our measure of fairness. In particular, Table S1 shows the descriptive statistics for the measures of autonomy and self-efficacy and S2 shows the questions asked for each measure. Tables S3 and S4 show descriptive statistics and the principal component analysis for fairness.

**S1 Table. Descriptive Statistics of Measures of Autonomy**

| Variable | Obs | Mean | Standard Deviation | Min | Max | Cronbach Alpha among components |
| --- | --- | --- | --- | --- | --- | --- |
| General Autonomy Index | 3,427 | 5.00 | 1.12 | 1.14 | 7.00 | 0.81 |
| General Self-Efficacy Index | 3,427 | 5.11 | 1.15 | 1.00 | 7.00 | 0.94 |

**S2 Table. Autonomy Measures**

| Measure | Scale for each question | Questions Included |
| --- | --- | --- |
| General Autonomy Index | 1 to 7 | I feel like I am free to decide for myself how to live my life. |
|  |  | I feel pressured in my life. |
|  |  | I generally feel free to express my ideas and opinions |
|  |  | In my daily life, I frequently have to do what I am told. |
|  |  | People I interact with on a daily basis tend to take my feelings into consideration |
|  |  | I feel like I can pretty much be myself in my daily situations. |
|  |  | There is not much opportunity for me to decide for myself how to do things in my daily life. |
| Self-Efficacy Index | 1 to 7 | I can always manage to solve difficult problems if I try hard enough |
|  |  | If someone opposes me, I can find the ways and means to get what I want. |
|  |  | I am certain that I can accomplish my goals |
|  |  | I am confident that I could deal efficiently with unexpected events |
|  |  | Thanks to my resourcefulness, I can handle unforeseen situations. |
|  |  | I can solve most problems if I invest the necessary effort. |
|  |  | I can remain calm when facing difficulties because I can rely on my coping abilities. |
|  |  | When I am confronted with a problem, I can find several solutions |
|  |  | If I am in trouble, I can think of a good solution |
|  |  | I can handle whatever comes my way. |

**S3 Table. Descriptive Statistics of Measures of Fairness**

| Variable | Obs | Mean | Std. Dev. | Min | Max | Cronbach Alpha among components |
| --- | --- | --- | --- | --- | --- | --- |
| 1st Principal Component Fairness | 3,427 | 0.00 | 2.29 | -3.71 | 7.04 | 0.87 |
| Troubled by income differences | 3,427 | 0.62 | 0.30 | 0.00 | 1.00 | - |
| Fair work hard and not afford decent living | 3,427 | 0.24 | 0.26 | 0.00 | 1.00 | - |
| Fair that there are billionaires | 3,427 | 0.32 | 0.31 | 0.00 | 1.00 | - |
| Fair to increase taxes extremely wealthy | 3,427 | 0.72 | 0.29 | 0.00 | 1.00 | - |
| Is US economic system fair | 3,427 | 0.45 | 0.29 | 0.00 | 1.00 | - |
| Unequal opportunities fair | 3,427 | 0.72 | 0.26 | 0.00 | 1.00 | - |
| Gender income inequality fair | 3,427 | 0.38 | 0.30 | 0.00 | 1.00 | - |
| Racial income inequality fair | 3,427 | 0.44 | 0.32 | 0.00 | 1.00 | - |
| Fair higher income buy better healthcare | 3,427 | 0.34 | 0.31 | 0.00 | 1.00 | - |
| Fair higher income buy better education | 3,427 | 0.37 | 0.30 | 0.00 | 1.00 | - |

**S4 Table. Principal Components of Measures of Fairness**

| **Component** | **Eigenvalue** | **Difference** | **Proportion** | **Cumulative** |
| --- | --- | --- | --- | --- |
| Comp1 | 5.26 | 4.32 | 0.53 | 0.53 |
| Comp2 | 0.94 | 0.18 | 0.09 | 0.62 |
| Comp3 | 0.75 | 0.18 | 0.08 | 0.70 |
| Comp4 | 0.57 | 0.04 | 0.06 | 0.75 |
| Comp5 | 0.53 | 0.07 | 0.05 | 0.81 |
| Comp6 | 0.46 | 0.04 | 0.05 | 0.85 |
| Comp7 | 0.42 | 0.04 | 0.04 | 0.89 |
| Comp8 | 0.39 | 0.04 | 0.04 | 0.93 |
| Comp9 | 0.34 | 0.02 | 0.03 | 0.97 |
| Comp10 | 0.32 | . | 0.03 | 1.00 |

## Demographic Characteristics of People with High and Low Autonomy

As indicated in the main text, the following table presents demographic characteristics of the people with low and high autonomy in our sample, as determined by a median split of the General Index of Autonomy.

**S5 Table. Demographic Characteristics by Levels of Autonomy**

|  | Low Autonomy | High Autonomy |
| --- | --- | --- |
| % Female | 54% | 56% |
| Median income | $50,000-54,999 | $55,000-59,999 |
| % White | 58% | 63% |
| % College education or more | 42% | 48% |
| Average Age | 41 | 52 |

## Autonomy and Comparison of Choice Availability

The following table shows the relationship between perceived autonomy and how many choices other people have compared to oneself. Column 1 presents the result for the question “Do you believe others have more or fewer choices than you have when it comes to finding and keeping a job they want?”  Column 2 presents the results for the question “Do you believe others have more or fewer choices than you have when it comes to selecting doctors, treatments, and medicines that they receive?” and column 3 for the question “Do you think others have more or fewer choices than you have when it comes to the schools they or their children attend?” In all cases, the dependent variable in the regression goes from 0 (others have many fewer choices) to 1 (others have many more choices).

**S6** **Table. Results of OLS Regression of Comparison of Choices on Autonomy**

|  | Dependent Variable: | | |
| --- | --- | --- | --- |
|  | Job Choice Comparison | Healthcare Choice Comparison | School Choice Comparison |
| General Index of Autonomy | -.005 | -.005 | .011* |
|  | (.004) | (.005) | (.004) |
| N | 3427 | 3427 | 3427 |
| R^2^ | .06 | .07 | .05 |
| Notes: *p<0.05, **p<0.01, ***p<0.001. Each column shows the unstandardized coefficient of the General Index of Autonomy in an OLS regression in which the variable at the top of the column is the dependent variable when including controls. Robust standard errors in parentheses. Controls for every column include age, perceived current and past economic status, and dummies for gender, race, state, income group, education level, and party ID. The range for the General Index of Autonomy is 1.14 to 7, with higher values indicating greater autonomy. | | | |
|  |  |  |  |
|  |  |  |  |
|  |  |  |  |

## Robustness Checks

In this section, we include analyses for other measures of inequality. In particular, we replicate Table 2 for the proportion of people in each of the three income brackets (Less than $45,000, between $45,000 and $135,000, and more than $135,000) without health insurance and without a college degree. Regardless of autonomy level, people underestimate the proportion of individuals without a college degree and overestimate the proportion without health insurance (the difference with the true quantity is always statistically significant at p<.01 level). Finally, the tables show that people with lower autonomy generally perceive greater inequality as they estimate lower proportions of people at the lower end of the income spectrum and higher proportions of people at the top of the income spectrum than those with higher autonomy. In Table S7, all of the differences between rows are statistically significant at p<.05 except between (a) and (b) in the “Between $45,000 and $135,000” category. In Table S8, the only non-statistically significant difference is between rows (a) and (b) in the “Less than $45,000” category.

**S7** **Table. Average Perceived and *True* Proportions without Health Insurance**

| \|  \| Less than $45,000 \| Between $45,000 and $135,000 \| More than $135,000 \| \| --- \| --- \| --- \| --- \| \| (a) Avg. High Autonomy \| 45.78 \| 31.36 \| 19.36 \| \| (b) Avg. Low Autonomy \| 48.82 \| 30.57 \| 16.60 \| \| (c) Census Bureau 2018 \| 13.90 \| 8.41 \| 3.58 \| \| Notes: The numbers from the Census Bureau are calculated for the entire population in 2018 using the American Community Survey Public Use Microdata Sample and personal weights. \| \| \| \| |
| --- | --- | --- | --- | --- | --- | --- | --- | --- | --- | --- | --- | --- | --- | --- | --- | --- | --- | --- | --- | --- |

**S8 Table. Average Perceived and *True* Proportions without College Degree**

| \|  \| Less than $45,000 \| Between $45,000 and $135,000 \| More than $135,000 \| \| --- \| --- \| --- \| --- \| \| (a) Avg. High Autonomy \| 51.85 \| 34.47 \| 25.19 \| \| (b) Avg. Low Autonomy \| 53.07 \| 33.07 \| 23.25 \| \| (c) Census Bureau 2018 \| 84.53 \| 68.29 \| 40.62 \| \| Notes: The numbers from the Census Bureau are calculated for the entire population in 2018 using the American Community Survey Public Use Microdata Sample and personal weights. \| \| \| \| |
| --- | --- | --- | --- | --- | --- | --- | --- | --- | --- | --- | --- | --- | --- | --- | --- | --- | --- | --- | --- | --- |

Tables S9 and S10 present similar results to those in tables S7 and S8 but using a continuous measure of the General Index of Autonomy and including controls in a linear regression. The tables show a similar pattern to that reported in Table 3 in the main text, but with more muted results.

**S9 Table. OLS Regression Results of Perceptions of Health Insurance on Autonomy**

|  | Dependent Variable | | |
| --- | --- | --- | --- |
|  | Percent <$45k w/o insurance | Percent $45k-$135k w/o insurance | Percent >$135k w/o insurance |
| General Index of Autonomy | -1.009* | -.539 | .485 |
|  | (.468) | (.359) | (.441) |
| DV Average | 47.33 | 30.96 | 17.95 |
| N | 3427 | 3427 | 3427 |
| R^2^ | .05 | .04 | .04 |
| Notes: *p<0.05, **p<0.01, ***p<0.001. Each column shows the unstandardized coefficient of the General Index of Autonomy in an OLS regression in which the variable at the top of the column is the dependent variable when including controls. Robust standard errors are in parentheses. Controls for every column include age, perceived current and past economic status, and dummies for gender, race, state, income group, education level, and party ID. The range for the General Index of Autonomy is 1.14 to 7, with higher values indicating greater autonomy. As a reference, the table also reports the average value of the dependent variable. | | | |

**S10 Table. OLS Regression Results of Perceptions of College Attainment on Autonomy**

|  | Dependent Variable | | |
| --- | --- | --- | --- |
|  | Percent <$45k w/no college attainment | Percent $45k- 135k w/no college attainment | Percent >$135k w/no college attainment |
| General Index of Autonomy | -.650 | .049 | .086 |
|  | (.475) | (.296) | (.416) |
| DV Average | 52.47 | 33.76 | 24.2 |
| N | 3427 | 3427 | 3427 |
| R^2^ | .05 | .07 | .03 |
| Notes: *p<0.05, **p<0.01, ***p<0.001. Each column shows the unstandardized coefficient of the General Index of Autonomy in an OLS regression in which the variable at the top of the column is the dependent variable when including controls. Robust standard errors are in parentheses. Controls for every column include age, perceived current and past economic status, and dummies for gender, race, state, income group, education level, and party ID. The range for the General Index of Autonomy is 1.14 to 7, with higher values indicating greater autonomy. As a reference, the table also reports the average value of the dependent variable. | | | |
|  |  |  |  |

Finally, as discussed in the main text, Table S11 presents the same results as Table 3 but with the Generalized Self Efficacy Index as the main independent variable. Results go in the same direction, but are smaller in size that the General Index of Autonomy.

**S11 Table. OLS Regression Results of Inequality on Self-Efficacy**

|  | Less than $45,000 | Between $45,000 and $135,000 | More than $135,000 | Are there large income differences? |
| --- | --- | --- | --- | --- |
| General Self-Efficacy Index | -.403 | .145 | .258 | -.007* |
|  | (.305) | (.258) | (.227) | (.003) |
| DV Average | 43.03 | 38.55 | 18.41 | 0.96 |
| N | 3427 | 3427 | 3427 | 3427 |
| R^2^ | .10 | .13 | .07 | .03 |
| Notes: *p<0.05, **p<0.01, ***p<0.001. Each column shows the unstandardized coefficient of on the General Self-Efficacy Index in an OLS regression in which the variable at the top of the column is the dependent variable when including controls. Robust standard errors are in parentheses. Controls for every column include age, perceived current and past economic status, and dummies for gender, race, state, income group, education level, and party ID. The range for the General Self-Efficacy Index is 1 to 7, with higher values indicating greater autonomy. As a reference, the table also reports the average value of the dependent variable. | | | | |
|  |  |  |  |  |
|  |  |  |  |  |
